# Current paradigm and futuristic vision on new-onset diabetes and pancreatic cancer research

**DOI:** 10.3389/fphar.2025.1543112

**Published:** 2025-05-23

**Authors:** Russell Moreland, Abigail Arredondo, Anupam Dhasmana, Swati Dhasmana, Shabia Shabir, Asfia Siddiqua, Bonny Banerjee, Murali M. Yallapu, Stephen W. Behrman, Subhash C. Chauhan, Sheema Khan

**Affiliations:** ^1^ Department of Immunology and Microbiology, School of Medicine, University of Texas Rio Grande Valley, McAllen, TX, United States; ^2^ South Texas Center of Excellence in Cancer Research, School of Medicine, The University of Texas Rio Grande Valley, McAllen, TX, United States; ^3^ Department of Computer Science & Engineering, National Institute of Technology, Srinagar, India; ^4^ Institute for Intelligent Systems, and Department of Electrical and Computer Engineering, University of Memphis, Memphis, TN, United States; ^5^ Department of Surgery, Baptist Memorial Medical Education, Memphis, TN, United States

**Keywords:** pancreatic cancer, new-onset diabetes, screening strategies, biomarker discovery, socio-economic factors

## Abstract

New-onset diabetes (NOD) has emerged as a potential early indicator of pancreatic cancer (PC), necessitating a refined clinical approach for risk assessment and early detection. This study discusses critical gaps in understanding the NOD-PC relationship and proposes a multifaceted approach to enhance early detection and risk assessment. We present a comprehensive clinical workflow for evaluating NOD patients, incorporating biomarker discovery, genetic screening, and AI-driven imaging to improve PC risk stratification. While existing models consider metabolic factors, they often overlook germline genetic predispositions that may influence disease development. We propose integrating germline genetic testing to identify individuals carrying pathogenic variants in cancer-susceptibility genes (CSGs), enabling targeted surveillance and preventive interventions. To advance early detection, biomarker discovery studies must enroll diverse patient populations and utilize multi-omics approaches, including genomics, proteomics, and metabolomics. Standardized sample collection and AI-based predictive modeling can refine risk assessment, allowing for personalized screening strategies. To ensure reproducibility, a multicenter research approach is essential for validating biomarkers and integrating them with clinical data to develop robust predictive models. This multidisciplinary strategy, uniting endocrinologists, oncologists, geneticists, and data scientists, holds the potential to revolutionize NOD-PC risk assessment, enhance early detection, and pave the way for precision medicine-based interventions. The anticipated impact includes improved early detection, enhanced predictive accuracy, and the development of targeted interventions to mitigate PC risk.

## Introduction

Pancreatic cancer (PC) is among the deadliest solid malignancies. The National Cancer Institute estimated 62,210 new cases in 2022, with a projected 49,830 deaths (Chuong et al., 2023). Most patients are diagnosed at an advanced stage, with either local invasion or metastasis. Only 11% of cases are identified at a localized stage, where the 5-year survival rate is 39%. Across all stages, the overall 5-year survival rate remains low, at just 10% from the time of diagnosis (Baydogan et al., 2024). The endocrine part of the pancreas, which produces hormones like insulin and glucagon, plays a key role in glucose metabolism, while the exocrine pancreas produces digestive enzymes. The majority of pancreatic cancers arise from the exocrine tissue, specifically pancreatic ductal adenocarcinoma (PDAC), which is the most common and aggressive form (Backx et al., 2022). Early precursor lesions, such as pancreatic intraepithelial neoplasia (PanIN), intraductal papillary mucinous neoplasms (IPMNs), and mucinous cystic neoplasms (MCNs), play a crucial role in PC development and progression (Distler et al., 2014). Among these, PDAC has the strongest correlation with new-onset diabetes (NOD), as tumor-related metabolic disruptions can drive insulin resistance and β-cell dysfunction.

The treatment outcome and management of pancreatic cancer (PC) can be significantly improved if diagnosed either at a very early stage or by screening a high-risk population before clinical presentation of the disease. Research on these factors is crucial as pancreatic cancer cases are rapidly increasing and are projected to become the second leading cause of cancer-related deaths in the United States by 2030 (Luo et al., 2023; An et al., 2024). Thus, the identification of early detection molecular signatures, predictor of disease occurrence and/or socio-behavioral risk factors is highly desirable in PC field to reduce exceptionally high mortality rate posed by this disease. In the United States, diabetes is a growing public health issue, with an increasing proportion of cases classified as new-onset diabetes (NOD) (Klonoff, 2009). Recent studies suggest that NOD may be a potential indicator of PC, but the relationship between the two remains complex and poorly understood (Sharma et al., 2018; Trembath et al., 2024; Yang et al., 2024; Chandra et al., 2025). The risk of developing NOD increases significantly in individuals over 50 years old (Nguyen et al., 2012). From a public health perspective, both pancreatic cancer and diabetes impose a significant burden on healthcare systems. Diabetes affects over 37 million Americans, with an increasing number of cases classified as New-onset in older adults. Given that pancreatic cancer is projected to become the second leading cause of cancer-related deaths in the United States by 2030, identifying high-risk individuals through NOD screening could transform early detection strategies (Park et al., 2021). This study aims to bridge the critical research gap by emphasizing the intersection of diabetes and pancreatic cancer as a key opportunity for early intervention. Addressing this issue through multidisciplinary collaboration, healthcare policy updates, and public awareness initiatives is essential to reducing mortality and improving patient prognosis.

Pancreatic cancer rates have increased with age, especially in older individuals with Type 2 diabetes Mellitus (T2DM) due to shared metabolic and inflammatory pathways. Surprisingly, the risk of pancreatic cancer was inversely associated with the age at which T2DM began. Remarkably, a higher Standardized Incidence Ratio (SIR) of 5.73 (95% CI, 4.49-7.22) was observed in the 20-54 age group (Shen et al., 2023). Emerging as a significant area of research, NOD poses a rising concern, especially among middle-aged and older individuals. Amidst various factors contributing to diabetes development, recent studies propose NOD as a potential early indicator of PC. Although type 2 diabetes is established as a PC risk factor, it's also suggested that diabetes could be an early symptom of an underlying pancreatic tumor (Huxley et al., 2005; Ben et al., 2011; Li et al., 2012). PC’s low 5-year survival rate of 7.7% is attributed to late-stage diagnosis (Patel et al., 2014; Liao et al., 2019) emphasizing the need for early detection. Lacking routine screening, understanding the connection between NOD and PC is gaining traction as a critical focus for improved patient outcomes.

The intricate link between NOD and PC remains enigmatic, yet delving into this connection offers potential for improved screening tactics and early PC detection. Pancreatic cancer-associated diabetes (PCDM) constitutes about 1% of NOD cases, with almost half of PC patients succumbing within 2 years before official diagnosis (Liao et al., 2019). PCDM refers to a form of diabetes that develops in patients with pancreatic cancer, often as a result of tumor-induced changes in pancreatic function. While type 2 diabetes has been recognized as a risk factor for PC in epidemiologic studies (Pannala et al., 2008), there is also evidence that diabetes may be a manifestation of the pancreatic tumor. Therefore, investigations are warranted to affirm whether diabetes can be a risk factor and an early symptom of PC or a consequence of PC. Studies have shown links between high prevalence of NOD among PC cases, and that glucose metabolism improves after tumor resection (Pannala et al., 2008; Andersen et al., 2017). The relationship between NOD and pancreatic malignancy has been explored in mainly case-control studies (Huang et al., 2020a) or within cohorts of primarily white individuals (Gupta et al., 2006; Huang et al., 2020a). Furthermore, many of the cohort studies consisted solely of diabetes patients and could only report a standardized incidence ratio because they lacked data on a non-diabetes comparison group (Adami et al., 1991; Chow et al., 1995; Jee et al., 2005). A study showed that NOD in African American and Hispanic men and women over age 50 is associated with an early indication of PC citation (Setiawan et al., 2018). Other than these latest findings, however, research in ethnically diverse populations is quite limited. This type of study can be very useful to precisely determine the etiology of PC and for developing new diagnostic and/or prognostic biomarkers of PC.

In this article, we have reviewed the findings of all benchmarks conducted and investigated the candidate markers that are altered during PC and DM using pathway enrichment analysis. The existing literature on the relationship between NOD and PC is reviewed, with a focus on the implications for refining screening strategies. The article examines the potential for biomarker discovery and machine learning approaches to improve screening accuracy and discusses the importance of addressing socio-economic and behavioral factors that may contribute to disease risk. Additionally, the article suggests key focus areas for future research, including investigating the relationship between NOD and pancreatic malignancy in ethnically diverse populations. By understanding the complex relationship between NOD and PC, more effective screening strategies can be developed, potentially improving patient outcomes and saving lives. Finally, we also discuss possible solutions to address these factors as well as other factors that have not yet been investigated but can be benchmarked in the future.

## Relevance of NOD in early detection of PC: lessons learnt so far

Several investigations have delved into the potential of early PC detection in individuals with NOD, though the precise connection between the two remains elusive. Although some research suggests NOD could be a potential risk factor for pancreatic cancer, the interplay of diabetes with other conditions such as myocardial infarction indicates a more complex relationship. Furthermore, the close occurrence of NOD and PC within a relatively short span hints at NOD possibly being an outward result of PC. Additionally, the abrupt diabetes onset in PC patients has been correlated with heightened cancer aggressiveness. Despite the relatively low 3-year PC risk of 0.11% in individuals over 50, this risk rises to 0.9% in those with NOD (Sharma et al., 2018). Further research exploring the molecular association between NOD and PC could help clarify the relationship between these two conditions and improve risk prediction models.

The relationship between developing NOD and a subsequent diagnosis of PDAC remains a significant area of research. Worldwide, researchers are striving to create more accurate prediction models using various clinical data points. For instance, a study from Korea found that men over 50 years old who were recently diagnosed with NOD had an increased risk of developing PDAC, with a hazard ratio of 7.45 (Lee et al., 2023). Similarly, a Danish study found that 0.6% of patients over 50, regardless of sex, who were recently diagnosed with NOD developed PDAC (Jensen et al., 2023). In England, researchers developed a model with good predictive accuracy for patients with NOD who later developed PDAC, with the highest 1% of predicted risk capturing 12% of cases (Clift et al., 2024). Additionally, an Australian study improved their predictive models for patients diagnosed with NOD who would develop PDAC, achieving a PPV of 1.3 (Ali et al., 2024). The ongoing interest in developing more accurate predictive models highlights the importance of discovering new biomarkers to enhance early intervention in patients with PDAC.

Many studies suggest that new-onset diabetes (NOD) can be a helpful indicator for the early detection of PC. The Enriching NOD for pancreatic cancer (ENDPAC) model is one example of a risk prediction model that has been proposed and validated to identify individuals with NOD who may be at higher risk for PC (Sharma et al., 2018). The ENDPAC model takes into account factors such as age, weight loss, and rise in blood glucose in the year before NOD to assess risk. Studies have found that a significant proportion of PC cases occur within 12 months of NOD onset (Kikuyama et al., 2018). While there are currently no official biomarkers for PC, some laboratory evidence suggests that PC cells can produce molecules that affect glucose metabolism and cause hyperglycemia, supporting the potential of NOD as a clue for early detection (Maitra and Chari, 2019). As the incidence of PC is on the rise, understanding the transformation from a healthy state to prediabetes/diabetes and PC is crucial. Laboratory studies indicate that PC cell line supernatants can cause beta cell malfunction and produce soluble molecules that alter glucose metabolism, leading to hyperglycemia *in vitro.* Additionally, PC exosomes are responsible for the paraneoplastic dysfunction of human beta-cells and inhibit insulin secretion. (Maitra and Chari, 2019; Mizuno et al., 2020). Additionally, some studies have found a potential link between NOD and the development of intraductal papillary mucinous neoplasm (IPMN), which can progress to PC (Leal et al., 2015; Sousa et al., 2020). Therefore, these studies support NOD to be a clue to detect PC in the early stage and improve the prognosis of this intractable malignancy.

The connection between the emergence of type 2 diabetes and PC is associated with beta-cell dysfunction within the pancreas, leading to an impaired response in insulin secretion triggered by glucose (Ueda et al., 2009). Furthermore, the duration of diabetes contributes to the likelihood of PC development. Additionally, the length of time a person has had diabetes impacts the risk of PC. Presently, the DETECT study is investigating the distinct pathophysiology and specialized biomarkers of PC and diabetes, employing a mixed meal test to distinguish between type 2 diabetes and pancreatogenic diabetes (Gallo et al., 2021). These risk prediction models aim to enhance early detection of PC by utilizing early symptoms in patients with a higher risk level. Recent evidence demonstrates that diabetic patients with a duration of 1–4 years have a higher risk of PC compared to diabetic patients with a duration of 5–9 years (Muniraj and Chari, 2012). The relative risk factor is 1.4924 and significantly higher (5.38) in diabetic patients with less than 1 year of diabetes diagnosis (Muniraj and Chari, 2012). Furthermore, persistent diabetes could also pose a risk for PC, given the shared presence of insulin resistance in both conditions.

The shared symptoms of PC and diabetes contribute to the heightened risk of PC development. Research indicates that cancer cells might trigger abnormal beta-cell function, causing diabetes mellitus, and resulting in a notably elevated PC risk in individuals with new-onset diabetes compared to those with long-standing diabetes (Gallo et al., 2021). PCDM is characterized by beta-cell malfunction and marked peripheral insulin resistance, and its pathophysiology remains unclear (Liao et al., 2019). Research findings have demonstrated a connection between prolonged diabetes and the emergence of pancreatic exocrine neoplasia and uncontrolled tumor proliferation. Furthermore, a clinical study involving a cohort of patients has indicated that new-onset diabetes might contribute to the advancement of tumors in individuals with PC (Dai et al., 2016). As the symptoms of PC are often shared with diabetes, the asymptomatic phase can disguise the presence of PC in about 80% of patients [24]. PC cells secrete tumor-inducing factors, such as adrenomedullin, leading to an increase in insulin resistance and beta cell dysfunction in PCDM (Aggarwal et al., 2012; Dai et al., 2016).

Elderly individuals with NOD face a significantly elevated risk, six to eight times higher of developing PC (PC) compared to the general population, rendering this group particularly susceptible to rapid PC onset (Singhi et al., 2018; Gallo et al., 2021). However, there are currently no established guidelines for preventing PC in high-risk NOD patients (Pereira et al., 2020; Gallo et al., 2021). While new-onset diabetes is frequently linked to pancreatic cancer, research indicates that it can resolve following resection, a viable option for only a minority of patients (Ben et al., 2012). Therefore, people with NOD require closer monitoring and attention. Interestingly, long-standing diabetes was found to be strongly associated with PC in Latinos at the age of 75, but not in African Americans. African Americans and Latinos are two minority groups with the highest diabetes risk but with differing PC rates (Setiawan et al., 2019b). While indications propose that NOD might manifest due to PC, ongoing research is exploring this connection, which could potentially serve as an early indicator for PC detection. If successful, this advancement in diabetes and PC research could provide answers and potentially enable earlier identification of this fatal ailment.

## Identification of genomic alterations and pathways associated with the co-occurrence of pancreatic cancer and diabetes through data mining

**FIGURE 1:**
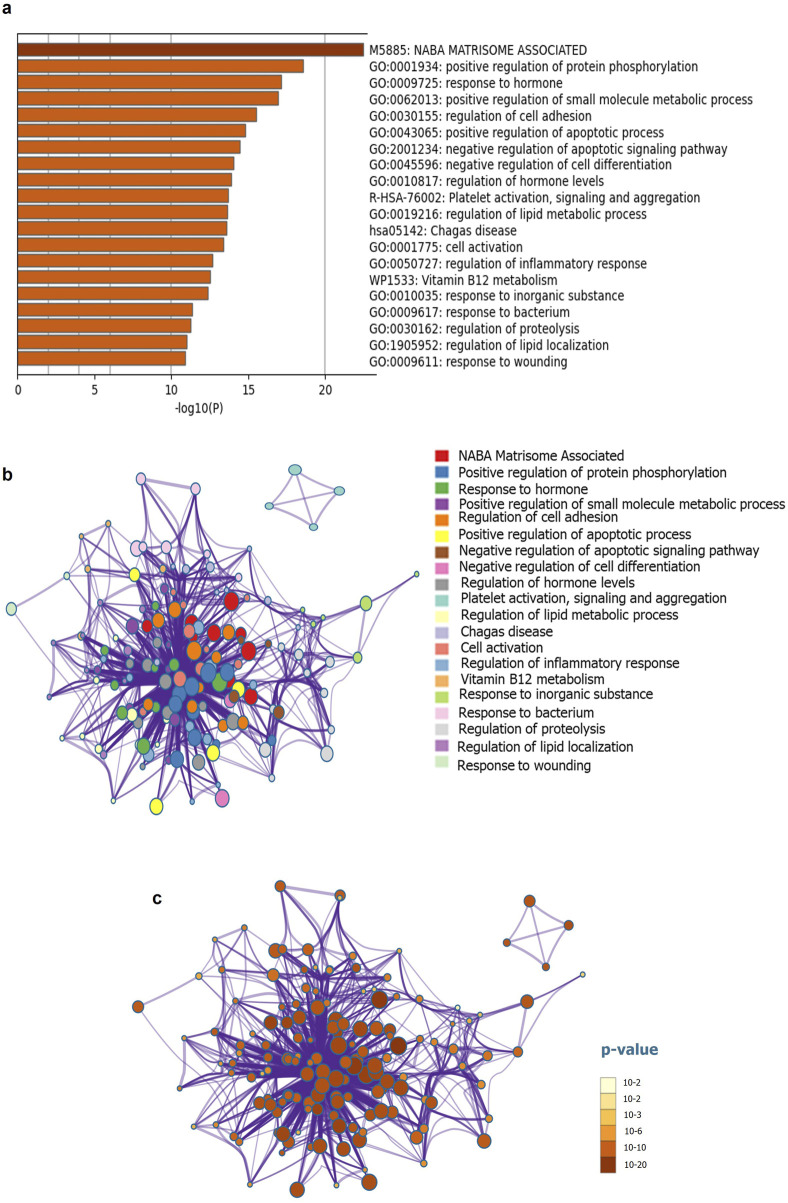
Functional enrichment and interactome analysis **(a)** Metascape bar graph for viewing top non-redundant enrichment clusters **(b)** Metascape visualization of the interactome network for viewing 20 enriched pathways **(c)** Metascape visualization for enriched pathways, using a discrete color scale to represent statistical significance.

The discovery of biomarkers has led to improved diagnosis of PC at the biomolecular level. In this review, we have summarized the results of previous studies and investigated candidate markers that are altered during pancreatic cancer-associated diabetes mellitus (PA-DM) and/or NOD. Using a PubMed literature survey, we retrieved data related to 75 proteins associated with PC-associated PA-DM or NOD, using keywords such as PC, PC, pancreatitis, diabetes, diabetes mellitus, type 2 diabetes, NOD, and type 3c diabetes mellitus. All proteins were listed in Supplementary Table 1 with their respective UniProt IDs. Furthermore, we provide insight into the molecular network using pathway enrichment analysis and constructed a protein interactome network (PIN) using GeneMania (Figure 1; Supplementary Figure S1). Interaction selection was based on co-expression, co-localization, physical interaction, shared protein domains, and genetic interactions related features (Warde-Farley et al., 2010). To identify genetic alterations and pathways involved in the co-occurrence of PC and diabetes, we conducted a thorough review of existing studies and retrieved data on 75 proteins associated with PA-DM or NOD. The proteins were curated manually through a literature survey using relevant keywords (Supplementary Table 1). We listed these proteins in Supplementary Table 2 with their corresponding UniProt IDs and conducted pathway enrichment analysis using Metascape (Figures 1A,B). Our analysis revealed 20 enriched pathways with significant p-values ranging from 10^−2^ (p < 0.01) to 10^−20^ (p < 0.05 or higher) (Zhou et al., 2019) (Figure 1C). Further mining of the enrichment data identified genetic alterations occurring in matrisome genes, which encode extracellular matrix (ECM) proteins and modulate ECM structure and function. The accumulation of ECM, referred to as desmoplasia, is a pathological characteristic and key contributor to PC (Suklabaidya et al., 2018). Hyperglycemia increases desmoplasia and leads to alterations in the ECM, resulting in increased production of matrix metalloproteinases (MMPs) (Henke et al., 2019; Huang and Kyriakides, 2020). The higher ECM content in tumors can explain why metformin can reduce desmoplasia in PC by regulating blood sugar levels (Incio et al., 2015).

Our analysis suggests that increased protein phosphorylation plays a role in the initiation and aggressiveness of PC during PA-DM. One example of this is the protein Interleukin-4 (IL-4) which, when used to treat cells that overexpress the protein insulin receptor substrate 1 (IRS-1), leads to rapid phosphorylation of IRS-1. IRS-1 is an important adaptor in insulin and insulin-like growth factor signaling, which is involved in the downstream effector of KRAS, PI3K/AKT/mTOR signaling pathway, and is crucial in regulating key cancer hallmarks (Awasthi et al., 2012; Mortazavi et al., 2022). Activation of IRS-1 expression through phosphorylation increases the proliferation, invasion, and migration abilities of PC cells (Huang et al., 2018). While the impact of IRS-1 on insulin resistance and diabetes is well-documented, its connection to PC remains incompletely understood. Hyperglycemia is often accompanied by metabolic and molecular changes that can affect cancer risk, progression, treatment, and mortality (Huang et al., 2020b). Our analysis further highlights significant changes in metabolic genes associated with both non-obese diabetic NOD and PA-DM. In type 2 diabetes, chronic hyperglycemia results from both pancreatic beta cells that are unresponsive to insulin secretion and insulin resistance in target tissues (Ouni et al., 2020). If T2DM is prolonged and high levels of lipids and glucose are reached, pancreatic islet function declines, leading to insufficient compensation and beta cell loss (Ouni et al., 2020). Before a diagnosis of diabetes or associated symptoms like weight loss occur, individuals with NOD who develop PC are found to have significantly higher levels of glucose and HbA1c. However, it is important to consider racial and ethnic differences in cancer risk when examining the impact of glycemic parameters on cancer risk, as this was not accounted for in the referenced study (Huang et al., 2020b).

A different study has suggested that African Americans and Latinos with NOD exhibit higher metabolic markers, increasing their risk for PC when compared to those with long-standing diabetes in the Multiethnic Cohort (Huang et al., 2020b; Lemanska et al., 2022). NOD diabetes was associated with a significantly higher risk of pancreatic cancer in African Americans and Latinos, with a 4.08-fold increased risk in Latinos and a 3.38-fold increased risk in African Americans, supporting its role as an early manifestation of pancreatic cancer (Setiawan et al., 2019a). South Asians and Pacific Islanders demonstrate higher rates of insulin resistance and type 2 diabetes, which could alter pancreatic cancer risk patterns in these populations (Shah and Kanaya, 2014). Additionally, research from the National Health and Nutrition Examination Survey (NHANES) has shown that underserved communities often experience delayed diagnoses and limited access to advanced screenings, further exacerbating disparities in pancreatic cancer outcomes. Additionally, New-onset diabetes was associated with a nearly sevenfold increased risk of pancreatic cancer across Asian, Black, Hispanic, and White populations, with weight loss and rapid worsening of glycemic control serving as early metabolic indicators of elevated risk (Huang et al., 2020b). To address the gap in research on ethnically diverse populations, several avenues should be explored. Expanding large-scale multiethnic cohort studies can help clarify ethnic variations in the relationship between NOD and PC, leveraging databases such as the Multiethnic Cohort Study or the All of Us Research Program (Investigators, 2019; Bick et al., 2024). Additionally, genetic and molecular investigations focusing on ethnic-specific predispositions, insulin resistance patterns, and inflammatory biomarkers could identify key molecular pathways influencing PC risk (Supplementary Table 3). Community-based screening and early detection programs tailored for high-risk ethnic groups, including mobile health units and culturally adapted awareness campaigns, could improve early diagnosis and intervention (Kale et al., 2023). Furthermore, research on health disparities and socioeconomic factors, using public health databases like NHANES, is essential to understanding how systemic barriers contribute to delayed diagnosis and poorer outcomes. Addressing these gaps will improve our understanding of ethnic disparities in NOD-associated pancreatic cancer risk and contribute to better early detection and outcomes in underrepresented communities (Kale et al., 2023).

Changes in overall metabolic markers began to occur approximately 1.5–2 years before PC diagnosis, which roughly corresponds with the onset of diabetes (Perera and Bardeesy, 2015; Huang et al., 2020b). Further increases in blood glucose, weight loss, and serum lipids were observed within a few months (6-18) prior to diagnosis, with a noticeable rise occurring 6 months before diagnosis. This is when symptoms begin to appear in many patients due to the neoplastic disorder caused by tumor cells secreting molecules, such as adrenomedullin (AM), which inhibits insulin secretion by β-cells (Aggarwal et al., 2012). Patients with PC were found to have higher levels of AM, leading to glucose tolerance in PC mice with PC. AM receptors upregulate p38 and ERK1/2 MAPKs, promoting lipolysis and β-cell dysfunction [45]. Additionally, calcitonin gene-related peptide (CGRP) receptors have been recently found to be critically important for metabolism, vascular tone, and inflammatory response (Aggarwal et al., 2012). However, given that T2DM is much more common than NOD in PC cases, further biomarkers are necessary to differentiate between the conditions to arrive at meaningful conclusions (Liao et al., 2019). Individuals with PCDM show higher serum levels of galectin-3 and S100A9 compared to those with type 2 diabetes in the early stages of diabetes onset, suggesting that these biomarkers are produced by the pancreas and contribute to peripheral insulin resistance in PCDM (Liao et al., 2019). Additionally, a recent study has identified a serum miRNA profile that can significantly differentiate PC-associated NOD from non-cancer new-onset T2DM, consisting of six miRNAs: miR-483–5p, miR-19a, miR-29a, miR-20a, miR-24, and miR-25. This set of miRNAs could be further investigated as a potential biomarker for identifying NOD patients early and assessing their predisposition to PC (Dai et al., 2016). The study reports the first serum miRNA-based biomarker for distinguishing PCDM from non-cancer T2DM and the healthy group, while also providing insights into the progression of PCDM for further investigation (Dai et al., 2016).

Research has examined the impact of HMGN proteins on pancreatic beta cell function in mice. Findings indicate that HMGN3 is highly expressed in the islets of the pancreas, with gene expression levels distinctly higher in mouse islets compared to nearby exocrine cells, as confirmed by Western blot analysis (Ueda et al., 2009). HMGN3, a chromatin binding protein, may play a role in diabetes by influencing glucose regulation via its effects on GSIS in pancreatic beta cells [21]. The HMGN family of chromatin binding proteins is known for their dynamic modulation of epigenetic processes (Kugler et al., 2012). Using a prediction model, novel epigenetic alterations were identified in murine pancreatic islets during the prediabetic state with mild hyperglycemia before beta cell failure (Ouni et al., 2020). In individuals with prediabetes, 105 genes were found to be altered in blood cells, contributing to biomarkers for the onset of T2DM using DNA methylation (Ouni et al., 2020). These studies highlight the need for further investigations into the epigenetic mechanisms involved in T2DM development and the potential contribution of the HMGN family of chromatin binding proteins. HMGA2, a chromatin-binding protein from the HMGA family, drives tumor growth, metastasis, and chemoresistance in pancreatic cancer while also linked to diabetes due to its high expression in patients (Chiou et al., 2018).

While genetic predisposition plays a crucial role in the development of NOD and PC, many racial and ethnic groups are underrepresented in pharmacogenetic studies and clinical trials (Ortega and Meyers, 2014; Zou et al., 2023). This lack of representation limits the generalizability of current research findings and may contribute to disparities in care. Even as our understanding of the relationship between NOD-PC and its risk factors advances to a clinically significant level, significant barriers to effective screening and intervention remain. Patients from lower socioeconomic backgrounds or those living in underserved geographic areas often face challenges in accessing high-quality healthcare services, further widening disparities in outcomes (Ajilore et al., 2023). Though our goal is to identify early risk factors for NOD-PC, we must remain vigilant in addressing the persistent barriers to achieving a comprehensive understanding and ensuring effective implementation of interventions. This area of research remains open for further discoveries related to PC initiation or progression.

## Major focus areas towards risk stratification and development of screening strategies

To advance the understanding of the relationship between NOD (NOD) and pancreatic ductal adenocarcinoma (PC), investigations must focus on various aspects that determine the factors impacting this association. A key unsolved question in this area of research is whether the heightened PC risk associated with NOD is present across different racial or ethnic minorities. It may be valuable to investigate whether the metabolic profiles of incident diabetes patients show distinct patterns among specific racial or ethnic groups. These findings could provide greater insight into the complex relationship between diabetes and PC, enabling the tailoring of prevention techniques and the identification of patients most suitable for screening. The identification of biomarker-based screening strategies would further enable early cancer diagnosis in high-risk groups and the general population. Based on current observations and studies, the following approaches may be significant in identifying risk associations and stratifying molecular subtypes or highly vulnerable populations versus resilient ones.

### Establishing a biobank of ethnically diverse annotated biospecimens

To diagnose patients in the curable stage, a multicenter observational cohort study is crucial, and proper biobanking plays a critical role in facilitating this process. The limited availability of well-annotated biospecimens contributes to increased outliers and a lack of understanding in the field (Mendy et al., 2018). Therefore, it is necessary to include both population-based and disease-oriented biobanks. Population-based biobanks are significant and can collect biospecimens along with associated patient lifestyle, social, demographic, and environmental data from individuals (Annaratone et al., 2021). The specimens can be categorized as healthy individuals, NOD or NOD with PC. Healthy individuals can be randomly selected from the community or recruited as family members of patients with NOD and/or NOD with PC. These biobanks will facilitate investigations into the associations between NOD and PC risk, diagnosis, prognosis, and identifying potential predictive biomarkers of disease risk.

For disease-oriented biobanks, biospecimens can be categorized based on diagnostic and treatment information, and the biospecimen collection must include detailed demographic patient profiles, precise clinical annotations, treatment history, and accurate past health history. Data collection should include a questionnaire, clinical symptoms, body weight, fasting blood collection, and carbohydrate antigen 19-9. In addition to blood and oral swabs, other specimens such as juice (Yu et al., 2017; Suenaga et al., 2018), stool (Kisiel et al., 2012), urine (Arasaradnam et al., 2018; Blyuss et al., 2020) and pancreatic cyst fluid (Singhi et al., 2018) can be collected at every second visit to account for the influence of diet or lifestyle on the monitored parameters. These specimens have been shown to be attractive for demonstrating aberrant somatic mutations, microRNAs, or proteins (Ghamlouche et al., 2023). The study of NOD and PC in ethnically diverse populations is crucial for several reasons. Firstly, different ethnic groups may have varying susceptibilities to developing these conditions due to differences in genetic, environmental, and lifestyle factors. Studying diverse populations can provide insight into these differences and help identify unique risk factors for these diseases. Secondly, many studies on NOD and PC have predominantly focused on populations of European descent and may not be generalizable to other ethnic groups. This can result in disparities in screening, diagnosis, and treatment for non-European populations. Thirdly, understanding the complex relationship between NOD and PC in diverse populations can aid in the development of more effective screening and prevention strategies that are tailored to specific ethnic groups. This can improve early detection rates and ultimately lead to better outcomes for patients. Therefore, research on ethnically diverse populations is crucial for advancing our understanding of NOD and PC and developing effective strategies to detect and manage these conditions.

In the United States, several biobank initiatives have been established to support biomarker research. One prominent example is the All of Us Research Program, launched by the National Institutes of Health (NIH), which aims to collect biological samples and health data from a diverse population to advance precision medicine (Investigators, 2019; Bick et al., 2024). Additionally, the National Cancer Institute’s (NCI) Cancer Moonshot Biobank (Schuster et al., 2023), Pancreatic Cancer Early Detection Research Program (PCERP) biorepository and the Pancreatic Cancer Detection Consortium (PCDC) biorepository are other crucial initiatives that stores well-annotated biospecimens from PC patients, supporting biomarker discovery and early detection efforts. Moreover, academic institutions such as MD Anderson Cancer Center, South Texas and Mayo Clinic have established dedicated biobanks to facilitate pancreatic disease research by integrating clinical, genetic, and lifestyle data. We at the South Texas Center of Excellence in Cancer Research (ST-CECR), at UTRGV are developing a comprehensive biobank, which predominantly comprises a Hispanic/Latino population. These biobanks initiatives play a vital role in enhancing the availability of high-quality, well-annotated biospecimens, ultimately improving the identification of predictive biomarkers and advancing early diagnosis and treatment strategies for NOD and PC.

### Monitoring the PC ties between NOD and weight loss

Current guidelines do not approve screening of the general population, making it crucial to identify high-risk groups for whom the benefits of screening outweigh the risks. A long-term study of a cohort of 159,025 patients followed for 30 years revealed that sudden NOD accompanied by weight loss may indicate a high risk for PC. Individuals who experienced sudden weight loss (1–8 lbs) after recent NOD had more than three times the risk for PC (HR 3.61, 95% CI 2.14-6.10) compared to those without diabetes or weight loss. Those with sudden weight loss of more than 8 lbs had close to seven times the risk (HR 6.75, 95% CI 4.55-10.0) (Yuan et al., 2020). Therefore, large prospective studies for NOD and weight loss after age 50 years are necessary for PC surveillance. These conditions should be recognized by medical practitioners, especially in individuals who were previously fit/healthy and did not intentionally attempt to lose weight through diet or exercise. Surveillance is particularly warranted in a multiethnic diverse population to determine whether NOD and sudden weight loss are associated with a significant increase in risk for PC and whether this may be attributed to sporadic or familial associations. These studies should be conducted in a multicenter setting, considering the impact of geographic location on the study.

### Genetic and Metabolomic profiling to detect screening strategies for PC-NOD

The poor diagnostic strategies for PC lead to its high fatality rate. Early detection of hidden symptoms in NOD can potentially improve diagnosis and prevent patients from reaching late-stage cancer. Various methods such as models, screening tools, and non-invasive biomarkers can contribute to the early detection of asymptomatic PC. However, evidence supports that PC screening should only be considered for high-risk patients based on family genetic history. Screening at average risk levels is not recommended (Aslanian et al., 2020).

Screening for PC is often done at a later stage when surgical resection is no longer feasible (Poruk et al., 2013). The criteria and limitations for screening should be based on diseases associated with the development of PC. In high-risk patients with no pancreatic lesions present and NOD, a change in screening or surveillance interval may be necessary every 12 months (Aslanian et al., 2020). The lack of a simple screening method and the lengthy asymptomatic phase of PC contribute to its late diagnosis.

Obesity and NOD are risk factors that cause paraneoplastic DM due to the tumor, accounting for about 1% of newly diagnosed cases (Lang et al., 2021). Specific criteria are needed to distinguish regular diabetes from paraneoplastic DM in these high-risk patients (Lang et al., 2021). Developing effective screening strategies for PC-NOD requires a better understanding of the genetic and metabolic factors underlying this association. Identifying specific genetic mutations and metabolic pathways associated with PC-NOD can facilitate the development of non-invasive biomarkers and targeted therapies for early detection and prevention of PC. Like patients with germline mutations in PC predisposition genes, adults with NOD are considered high-risk PC or mucinous pancreatic cyst cohorts (Baydogan et al., 2024). Currently, glycemically defined NOD is the only high-risk PC for sporadic PC. Therefore, it is important to focus prospective biomarker validation efforts on high-risk cohorts before extending them to the general population. In addition, PC cases are known to have a familial predisposition, and it is of high interest to investigate the association of NOD cases with familial predisposition and germline mutations in known PC genes such as Hereditary nonpolyposis colorectal cancer, BRCA2 (breast-ovarian), PALB2, Familial atypical multiple mole melanoma (p16), Familial pancreatitis (PRSS1), Peutz-Jeghers (STK11/LKB1), and ATM. These studies will define the basis for their inheritance. Further comprehensive genomic analysis of the germline from PC-NOD patients will reveal unexpected germline mutations in known cancer predisposition genes, particularly in patients with a family history of other cancers.

Given that diabetes is a complex metabolic disorder, it is necessary to conduct serum metabolomics analysis and search for metabolic pathways associated with PC-related DM to establish a screening strategy for PC based on NOD. To establish a screening strategy for PC based on NOD and search for the metabolic pathways associated with PC-related type-3 DM, it is highly warranted to compare the serum metabolomic profiles of patients with NOD and those with Non-PC-NOD by liquid chromatography-mass spectrometry. Further criteria development for distinguishing between PC-NOD and Non-PC-NOD will assist in managing PC at its earlier stages in patients with NOD, providing a platform for the future development of an early detection protocol for sporadic or familial PC in NOD subjects that incorporates imaging and clinical parameters.

### Utilizing AI based image recognition tools for cancer prediction

Machine learning (ML) models are a promising class of models for early detection of PC in asymptomatic populations (Kenner et al., 2021). In the context of T2DM, researchers are exploring AI-based image recognition algorithms for predicting the risk of PC and managing patient care (Chan et al., 2020; Guermazi et al., 2022; Putra et al., 2022). Artificial Intelligence is currently being utilized to accurately interpret large data sets in an efficient and reproducible manner (Dias and Torkamani, 2019). AI-based image recognition tools have the potential to analyze abdominal CT scans for early detection of PC in patients with NOD (Kenner et al., 2021; Mendoza Ladd and Diehl, 2021). These tools can detect abnormal growths or changes in the size or shape of the pancreas by analyzing the images. By integrating various types of data, these tools can provide a more comprehensive understanding of an individual’s risk for developing cancer and enable personalized screening and prevention strategies.

The amalgamation of Principal Component Analysis (PCA) and Convolutional Neural Network (CNN) models in image analysis hold great promise in enhancing the accuracy and efficiency of image recognition tasks (Mehrabinezhad et al., 2024). By reducing the dimensionality of the dataset and identifying crucial features in the images, medical images can be analyzed more effectively. This process entails pre-processing the dataset of images for analysis by using PCA analysis to reduce the number of features. Subsequently, a CNN can be trained to extract key features from the images, such as edges and shapes. Finally, the extracted features can be utilized to classify the images using a separate machine learning algorithm. This approach can be particularly advantageous for large datasets with numerous features, as it can make subsequent analysis more efficient and precise. The combination of PCA and CNN models has the potential to transform image recognition tasks, including medical diagnosis, by enhancing the accuracy and efficiency of analysis. Ongoing research in this field is set to improve the effectiveness of this approach and lead to new applications for image recognition tasks. Several existing tools and preliminary applications support the integration of PCA and CNN models in medical image analysis. For instance, deep learning frameworks such as TensorFlow and PyTorch have been leveraged to implement CNN architectures combined with dimensionality reduction techniques like PCA, enhancing computational efficiency without compromising diagnostic accuracy (Liu et al., 2022; Liu et al., 2024). In medical imaging, DeepPCA-Net has demonstrated success in reducing noise and improving feature extraction in radiological scans, aiding in early disease detection. Additionally, studies on PCA-assisted CNNs in ultrasound videos, MRI and CT scan analysis have shown promising results in optimizing classification tasks while minimizing computational costs (Mehmood et al., 2023; Senthil et al., 2023; Sun et al., 2023; Wen et al., 2023). These advancements suggest that while challenges remain, particularly in clinical validation and scalability, the integration of PCA and CNN models is a practical approach with increasing adoption in real-world medical imaging applications. Future work should focus on refining these models through larger clinical datasets and validating their performance across diverse imaging modalities. The integration of PCA and CNN models is particularly advantageous when handling large datasets that contain numerous features, as it can enhance the effectiveness and precision of subsequent analysis. The amalgamation of these two models can result in improved accuracy and efficiency when conducting image recognition tasks, ultimately leading to enhanced diagnosis and treatment of various medical conditions.

## Workup for clinical assessment in NOD patients and enrolling them for biomarker discovery

**FIGURE 2:**
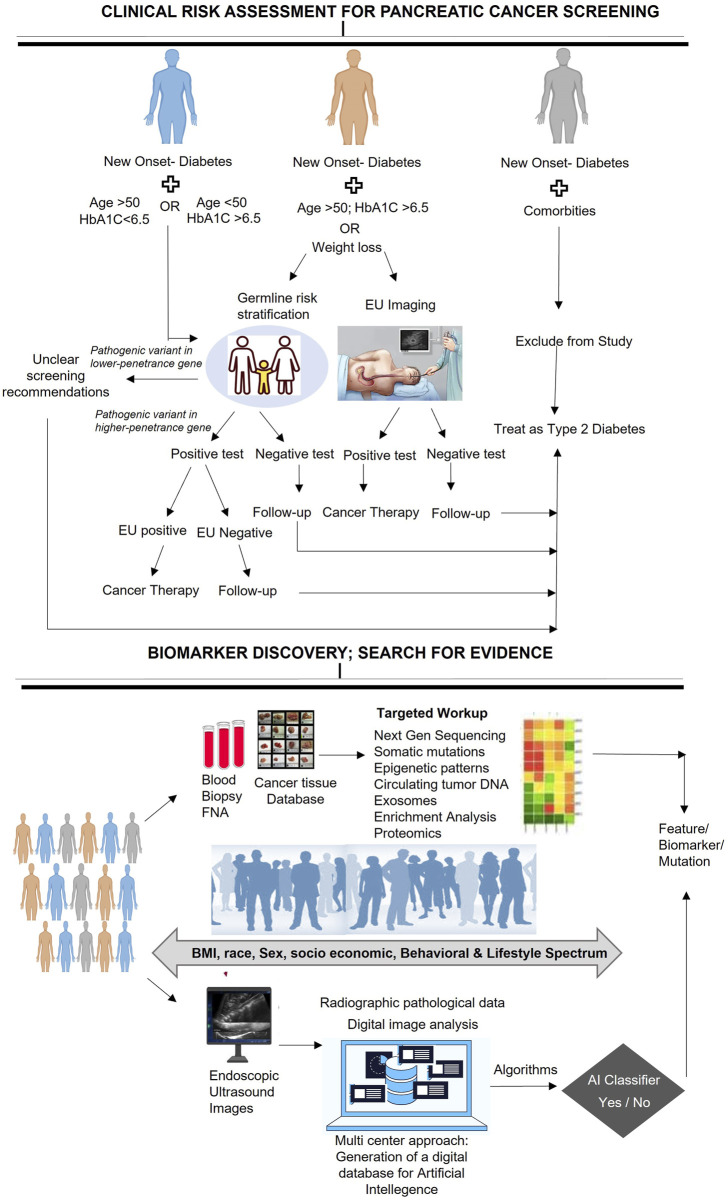
Clinical workflow of risk assessment for PC in NOD patients and biomarker discovery.

When conducting a clinical workup for patients with NOD, it is essential to perform a comprehensive assessment to identify any underlying medical conditions or risk factors that may be responsible for the development of diabetes. Furthermore, enrolling NOD patients in biomarker discovery studies can provide significant insights into the pathophysiology of diabetes and may pave the way for the development of novel diagnostic or therapeutic approaches. In this context, we present a clinical assessment workflow for patients diagnosed with NOD, which provides a stratified analysis to detect the risk of PC development. Although previous recommendations for PC screening in NOD patients, such as the ENDPAC model, have considered some vital parameters such as age at onset of diabetes, weight loss, and changes in glycemic index to predict PC risk, they have not taken into account germline risk stratification that could identify any genetic predispositions that may contribute to the development of NOD or other medical conditions (Sharma et al., 2018; Chen et al., 2021; Boursi et al., 2022; Hajibandeh et al., 2023). Therefore, we propose a workflow that outlines a systematic evaluation of NOD patients and provides stepwise follow-up directions to ensure a complete risk assessment of the patient (Figure 2) and offer appropriate guidance for further recommendations.

To enhance the clinical workflow for NOD patients, germline genetic testing can be included to identify those with pathogenic variants in cancer-susceptibility genes (CSGs), who are more prone to PC. Such testing can provide valuable information on the association of germline mutations with PC risk and NOD. A positive germline risk could help identify individuals who require special surveillance programs or prophylactic interventions to minimize the risk of PC cancer.

Enrolling NOD patients in biomarker discovery studies is a crucial step towards deepening our understanding of the underlying mechanisms that contribute to the development of PC in this patient population. By identifying novel molecular markers that may be relevant to NOD and PC, we can potentially develop new diagnostic or predictive tools for these conditions. To accomplish this, it is necessary to identify the population of interest, which may include individuals from diverse socioeconomic or demographic backgrounds, and to collect standardized samples such as blood, urine, or tissues for molecular profiling. Once the samples are collected, advanced techniques such as genomics, proteomics, or metabolomics can be used to identify potential biomarkers that may be influenced by socioeconomic, behavioral, or lifestyle factors. In addition, Endoscopic Ultrasound images can be analyzed to identify any abnormalities in the pancreas. The resulting data can then be processed using AI-based predictive models to identify individuals who may be at high risk for certain diseases or conditions. This approach may lead to the development of more personalized and precise screening and surveillance strategies for NOD patients, potentially leading to earlier detection of PC and improved patient outcomes. It may also help identify individuals who may benefit from targeted preventive interventions, including genetic counseling or lifestyle modifications, to reduce their risk of developing NOD and related conditions.

To ensure the accuracy and reproducibility of potential biomarkers identified through molecular profiling, it is essential to validate them in additional samples. Given the limited number of NOD patients available for study, conducting multicenter investigations is necessary to achieve conclusive results. A multicenter approach can increase sample size, recruit a diverse patient population, maintain uniform data collection and analysis, promote collaboration, and enhance the generalizability of the findings. Biomarker data should be combined with clinical information, such as age, gender, and medical history, to establish a comprehensive diagnostic or prognostic tool. The outcomes can be used to develop more efficient methods for identifying and treating patients with a higher risk of PC. Therefore, discovering biomarkers for risk stratification necessitates a multicentered approach that includes identifying potential biomarkers, integrating them with clinical information, constructing a predictive model, and testing the model. This approach will help us further our understanding of the relationship between these factors and the development of NOD and pancreatic diseases.

## Future perspectives and conclusion

In conclusion, the link between new-onset diabetes (NOD) and pancreatic cancer (PC) represents a critical frontier in medical research with profound clinical implications. While the precise mechanisms remain under investigation, mounting evidence suggests that individuals with NOD face a significantly higher risk of developing PC. This makes early detection in this high-risk group not just important, but essential for improving survival rates. Current research efforts are intensifying, focusing on identifying reliable biomarkers, advancing imaging technologies, and refining risk stratification tools to facilitate earlier diagnosis. Additionally, targeted lifestyle interventions and pharmacological strategies may offer promising avenues to mitigate PC risk in NOD patients.

To accelerate progress, collaboration between endocrinologists and oncologists must be prioritized to bridge the gap between metabolic dysfunction and tumor biology. A unified effort integrating endocrinology, oncology, genetics, and public health will be key to unraveling the molecular pathways linking NOD and PC. By leveraging cutting-edge technologies such as artificial intelligence and genomic profiling, risk-based clinical strategies can be revolutionized, paving the way for personalized, precision medicine approaches in screening and treatment.

Furthermore, identifying novel therapeutic targets through multidisciplinary research will be a game-changer in the fight against PC. Strengthening cross-specialty collaborations, particularly between endocrinologists and oncologists, will not only enhance early detection but also drive breakthrough innovations in intervention and care. By integrating these strategies, we move closer to transforming pancreatic cancer from a silent killer into a disease that can be detected earlier, treated more effectively, and ultimately prevented—offering new hope for high-risk individuals worldwide.

## Supplementary material

SUPPLEMENTARY FIGURE S1 Interactome analysis of curated gene set by using GeneMania.

SUPPLEMENTARY TABLE 1 Proteins deregulated in pancreatic ductal adenocarcinoma and diabetes mellitus.

SUPPLEMENTARY TABLE 2 Pathway and process enrichment analysis of input gene set.

SUPPLEMENTARY TABLE 3 PC risk in diverse ethnic groups.
