# Correction: Current paradigm and futuristic vision on new-onset diabetes and pancreatic cancer research

**DOI:** 10.3389/fphar.2025.1662270

**Published:** 2025-08-13

**Authors:** Russell Moreland, Abigail Arredondo, Anupam Dhasmana, Swati Dhasmana, Shabia Shabir, Asfia Siddiqua, Bonny Banerjee, Murali M. Yallapu, Stephen W. Behrman, Subhash C. Chauhan, Sheema Khan

**Affiliations:** ^1^Department of Immunology and Microbiology, School of Medicine, University of Texas Rio Grande Valley, McAllen, TX, United States; ^2^South Texas Center of Excellence in Cancer Research, School of Medicine, The University of Texas Rio Grande Valley, McAllen, TX, United States; ^3^Department of Computer Science & Engineering, National Institute of Technology, Srinagar, India; ^4^Institute for Intelligent Systems, and Department of Electrical and Computer Engineering, University of Memphis, Memphis, TN, United States; ^5^Department of Surgery, Baptist Memorial Medical Education, Memphis, TN, United States

**Keywords:** pancreatic cancer, new-onset diabetes, screening strategies, biomarker discovery, socio-economic factors

**Supplemental Figure S1** was omitted. The file has now been published.

The original version of this article has been updated.

