# Supplementary material for: Current paradigm and futuristic vision on new-onset diabetes and pancreatic cancer research
Source: Front Pharmacol. 2025 May 23;16:1543112. doi: 10.3389/fphar.2025.1543112 (PMC12141227; doi:10.3389/fphar.2025.1543112)
Supplement: Supplementary file 1 [file Table1.docx]

| **Supplementary Table:1** | **Proteins deregulated in Pancreatic Ductal Adenocarcinoma and Diabetes Mellitus** | | | | | |
| --- | --- | --- | --- | --- | --- | --- |
|  | **Protein Name** | **Gene Name** | **Ref.** | **PubMed ID** | **Uniprot ID** | **Remark** |
| 1 | Adrenomedullin | ADM | [1] | [22960655](https://pubmed.ncbi.nlm.nih.gov/22960655/) | [P35318](https://www.uniprot.org/uniprot/P35318) | Levels higher in PC-NOD; leads to insulin resistance in β-cells |
| 2 | Matrix metalloproteinase 9 | MMP9 | [2] | [20571492](https://pubmed.ncbi.nlm.nih.gov/20571492/) | [P14780](https://www.uniprot.org/uniprot/P14780) | Upregulated in PC-NOD |
| 3 | Vanin-1 (VNN1) | VNN1 | [2] | [20571492](https://pubmed.ncbi.nlm.nih.gov/20571492/) | [O95497](https://www.uniprot.org/uniprot/O95497) | Upregulated in PC-NOD |
| 4 | Osteoprotegerin | TNFRSF11B | [3] | [25641178](https://pubmed.ncbi.nlm.nih.gov/25641178/) | [O00300](https://www.uniprot.org/uniprot/O00300) | Upregulated in PC-NOD |
| 5 | S-100A8 N-terminal peptide* | S100A8 | [4] | [16678810](https://pubmed.ncbi.nlm.nih.gov/16678810/) | [P05109](https://www.uniprot.org/uniprot/P05109) | Upregulated in PC-NOD; leads to hyperglycaemia |
| 6 | Thrombospondin-1 (TSP-1) | THBS1 | [5] | [26573598](https://pubmed.ncbi.nlm.nih.gov/26573598/) | [P07996](https://www.uniprot.org/uniprot/P07996) | Decreased serum levels in PC-NOD |
| 7 | Betatrophin | ANGPTL8 | [6] | [27276680](https://pubmed.ncbi.nlm.nih.gov/27276680/) | [Q6UXH0](https://www.uniprot.org/uniprot/Q6UXH0) | Increased serum levels in PDAC-DM |
| 8 | Carcinoembryonic antigen 5 (CEA) | CEACAM5 | [7] | [20174821](https://pubmed.ncbi.nlm.nih.gov/20174821/) | [P06731](https://www.uniprot.org/uniprot/P06731) | Increased serum levels in PC-DM |
| 9 | Carbohydrate antigen 199 (CA-199) |  | [7] | [20174821](https://pubmed.ncbi.nlm.nih.gov/20174821/) | Not a protein | Increased serum levels in PC-DM |
| 10 | P38 MAPK* | MAPK11 | [8] | [27413117](https://pubmed.ncbi.nlm.nih.gov/27413117/) | [Q15759](https://www.uniprot.org/uniprot/Q15759) | Increased phosphorylation in PC-DM |
| 11 | Transforming Growth Factor β (TGF β)** | TGFBR1 | [9] | [32017070](https://pubmed.ncbi.nlm.nih.gov/32017070/) | [P36897](https://www.uniprot.org/uniprot/P36897) | Involved in signalling pathway which is involved in both DM and PDAC |
| 12 | Insulin-like growth factors 1 (IGF-1) | IGF1 | [10] | [19064563](https://pubmed.ncbi.nlm.nih.gov/19064563/) | [P05019](https://www.uniprot.org/uniprot/P05019) | Involved in DM development. Polymorphic variant in gene showed statistically significant risk for PDAC |
| 13 | Insulin-like growth factors 2 (IGF-2) | IGF2 | [10] | [19064563](https://pubmed.ncbi.nlm.nih.gov/19064563/) | [P01344](https://www.uniprot.org/uniprot/P01344) | Involved in DM development. Polymorphic variant in gene showed statistically significant risk for PDAC. |
| 14 | Connexin 26 | GJB2 | [11] | [15502644](https://pubmed.ncbi.nlm.nih.gov/15502644/) | [P29033](https://www.uniprot.org/uniprot/P29033) | Upregulated in patients with PDAC and glucose intolerance |
| 15 | Amylin/ Islet Amyloid Polypeptide | IAPP | [12] | [9428227](https://pubmed.ncbi.nlm.nih.gov/9428227/) | [P10997](https://www.uniprot.org/uniprot/P10997) | PDAC factor causes selective secretion of amylin |
| 16 | Interleukin-1 Receptor Antagonist (IL-1Ra) | IL1RN | [13] | [34990893](https://pubmed.ncbi.nlm.nih.gov/34990893/) | [P18510](https://www.uniprot.org/uniprot/P18510) | Increased plasma levels in patients with PDAC and NOD |
| 17 | Adiponectin | ADIPOQ | [13] | [34990893](https://pubmed.ncbi.nlm.nih.gov/34990893/) | [Q15848](https://www.uniprot.org/uniprot/Q15848) | Increased serum and plasma levels in patients with PDAC and NOD |
| 18 | Alpha 1-antichymotrypsin | SERPINA3 | [13] | [34990893](https://pubmed.ncbi.nlm.nih.gov/34990893/) | [P01011](https://www.uniprot.org/uniprot/P01011) | Increased serum and plasma levels in patients with PDAC-DM |
| 19 | C-Peptide |  | [13] | [34990893](https://pubmed.ncbi.nlm.nih.gov/34990893/) | ? | Increased serum levels in patients with PDAC-DM |
| 20 | Clusterin | CLU | [13] | [34990893](https://pubmed.ncbi.nlm.nih.gov/34990893/) | [P10909](https://www.uniprot.org/uniprot/P10909) | Decreased serum levels in patients with PDAC-DM |
| 21 | Ghrelin | GHRL | [13] | [34990893](https://pubmed.ncbi.nlm.nih.gov/34990893/) | [Q9UBU3](https://www.uniprot.org/uniprot/Q9UBU3) | Decreased serum levels in patients with PDAC-DM |
| 22 | Glucagon Like Peptide (GLP-1) | GCG | [13] | [34990893](https://pubmed.ncbi.nlm.nih.gov/34990893/) | [P01275](https://www.uniprot.org/uniprot/P01275) | Decreased serum levels in patients with PDAC-DM |
| 23 | Glucagon* |  | [13] | [34990893](https://pubmed.ncbi.nlm.nih.gov/34990893/) | ? | Decreased serum levels in patients with PDAC-DM |
| 24 | IFN-gamma | IFNG | [13] | [34990893](https://pubmed.ncbi.nlm.nih.gov/34990893/) | [P01579](https://www.uniprot.org/uniprot/P01579) | Increased plasma levels in patients with PDAC-DM |
| 25 | IL-4 | IL4 | [13] | [34990893](https://pubmed.ncbi.nlm.nih.gov/34990893/) | [P05112](https://www.uniprot.org/uniprot/P05112) | Increased plasma levels in patients with PDAC-DM |
| 26 | IL-6 | IL6 | [13] | [34990893](https://pubmed.ncbi.nlm.nih.gov/34990893/) | [P05231](https://www.uniprot.org/uniprot/P05231) | Increased plasma levels in patients with PDAC-DM |
| 27 | IL-7 | IL7 | [13] | [34990893](https://pubmed.ncbi.nlm.nih.gov/34990893/) | [P13232](https://www.uniprot.org/uniprot/P13232) | Increased plasma levels in patients with PDAC-DM |
| 28 | IL-8 | IL8 | [13] | [34990893](https://pubmed.ncbi.nlm.nih.gov/34990893/) | [P10145](https://www.uniprot.org/uniprot/P10145) | Increased plasma levels in patients with PDAC-DM |
| 29 | IL-9 | IL9 | [13] | [34990893](https://pubmed.ncbi.nlm.nih.gov/34990893/) | [P15248](https://www.uniprot.org/uniprot/P15248) | Increased plasma levels in patients with PDAC-DM |
| 30 | IL-12* | IL-12 | [13] | [34990893](https://pubmed.ncbi.nlm.nih.gov/34990893/) | ? | Increased plasma levels in patients with PDAC-DM |
| 31 | Insulin | INS | [13] | [34990893](https://pubmed.ncbi.nlm.nih.gov/34990893/) | [P01308](https://www.uniprot.org/uniprot/P01308) | Decreased plasma levels in patients with PDAC-DM |
| 32 | Leptin | LEP | [13] | [34990893](https://pubmed.ncbi.nlm.nih.gov/34990893/) | [P41159](https://www.uniprot.org/uniprot/P41159) | Decreased serum and plasma levels in patients with PDAC-DM |
| 33 | Macrophage inflammatory protein 1-alpha (MIP-1A) | CCL3 | [13] | [34990893](https://pubmed.ncbi.nlm.nih.gov/34990893/) | [P10147](https://www.uniprot.org/uniprot/P10147) | Increased plasma levels in patients with PDAC-DM |
| 34 | Macrophage inflammatory protein 1 (MIP-1B) | CCL4 | [13] | [34990893](https://pubmed.ncbi.nlm.nih.gov/34990893/) | [P13236](https://www.uniprot.org/uniprot/P13236) | Increased plasma levels in patients with PDAC-DM |
| 35 | Plasminogen activator inhibitor 1 (PAI-1) | SERPINE1 | [13] | [34990893](https://pubmed.ncbi.nlm.nih.gov/34990893/) | P05121 | Increase serum levels in patients with PDAC-DM |
| 36 | Platelet-derived growth factor subunit B (PDGF-B) | PDGFB | [13] | [34990893](https://pubmed.ncbi.nlm.nih.gov/34990893/) | [P01127](https://www.uniprot.org/uniprot/P01127) | Increase serum levels in patients with PDAC-DM |
| 37 | Regulated upon Activation, Normal T Cell Expressed and Presumably Secreted (RANTES) | CCL5 | [13] | [34990893](https://pubmed.ncbi.nlm.nih.gov/34990893/) | [P13501](https://www.uniprot.org/uniprot/P13501) | Increased patients with PDAC-DM |
| 38 | Von Willebrand factor (VWF) | VWF | [13] | [34990893](https://pubmed.ncbi.nlm.nih.gov/34990893/) | [P04275](https://www.uniprot.org/uniprot/P04275) | Increase serum levels in patients with PDAC-DM |
| 39 | Heparanase | HPSE | [14] | [31921662](https://pubmed.ncbi.nlm.nih.gov/31921662/) | [Q9Y251](https://www.uniprot.org/uniprot/Q9Y251) | In mice, was found to be activated by RAGE, and is overexpressed in PDAC |
| 40 | Transgelin-2 | TAGLN2 | [15] | [28521289](https://pubmed.ncbi.nlm.nih.gov/28521289/) | [P37802](https://www.uniprot.org/uniprot/P37802) | Upregulated in tissues of PDAC in a hyper insulinemic state |
| 42 | Sterol regulatory element-binding protein (SREBP)-1 | SREBF1 | [15] | [28521289](https://pubmed.ncbi.nlm.nih.gov/28521289/) | [P36956](https://www.uniprot.org/uniprot/P36956) | Upregulated in tissues of PDAC in a hyper insulinemic state |
| 43 | Peroxisome proliferator activated receptor-γ (PPARγ) | PPARG | [16] | [26937133](https://pubmed.ncbi.nlm.nih.gov/26937133/) | [P37231](https://www.uniprot.org/uniprot/P37231) | Regulates insulin metabolism in β-cells and may be protective to PDAC growth and metastasis. |
| 45 | Serine hydrolase carboxylesterase 2 (CES2) | CES2 | [17] | [35050739](https://pubmed.ncbi.nlm.nih.gov/35050739/) | [O00748](https://www.uniprot.org/uniprot/O00748) | Upregulated in tissue from mouse PDAC with T2DM |
| 46 | Dipeptidyl peptidase-IV (DPP-IV) | DPP4 | [18] | [27320722](https://pubmed.ncbi.nlm.nih.gov/27320722/) | [P27487](https://www.uniprot.org/uniprot/P27487) | Increased plasma activity levels in patients with PDAC and NOD or prediabetes |
| 47 | Fibroblast activation protein alpha (FAP) | FAP | [18] | [27320722](https://pubmed.ncbi.nlm.nih.gov/27320722/) | [Q12884](https://www.uniprot.org/uniprot/Q12884) | Decreased plasma activity level in patients with PDAC compared to T2DM, levels increased post-surgical resection |
| 48 | Regenerating gene I α (REG Iα) | REG1A | [19] | [20099282](https://pubmed.ncbi.nlm.nih.gov/20099282/) | P05451 | Increased expression in patients with PDAC-DM |
| 49 | Q motif containing GTPase activating protein 1 (IQGAP1) | IQGAP1 | [20] | [23639840](https://pubmed.ncbi.nlm.nih.gov/23639840/) | [P46940](https://www.uniprot.org/uniprot/P46940) | Proposed mediator in the mTORC1-Akt signalling pathway which may link DM t0 PDAC development |
| 50 | Glucose-dependent insulinotropic peptide (GIP) | GIP | [21] | [28027898](https://pubmed.ncbi.nlm.nih.gov/28027898/) | [P09681](https://www.uniprot.org/uniprot/P09681) | Decreased plasma levels in patients with PDAC and NOD/prediabetes |
| 51 | Pancreatic polypeptide (PP) | PPY | [21] | [28027898](https://pubmed.ncbi.nlm.nih.gov/28027898/) | [P01298](https://www.uniprot.org/uniprot/P01298) | Decreased plasma levels in patients with PDAC and NOD/prediabetes |
| 52 | Galectin-3 | LGALS3 | [22] | [31262951](https://pubmed.ncbi.nlm.nih.gov/31262951/) | [P17931](https://www.uniprot.org/uniprot/P17931) | Increased serum levels in patients with PDAC and NOD |
| 53 | Adenylyl cyclase 1 (Adcy1) | ADCY1 | [23] | [34512170](https://pubmed.ncbi.nlm.nih.gov/34512170/) | [Q08828](https://www.uniprot.org/uniprot/Q08828) | Target of PDAC released micrRNA-19a, may have role in insulin secretion dysregulation |
| 54 | Exchange protein directly activated by cAMP 2 (Epac2) | RAPGEF4 | [23] | [34512170](https://pubmed.ncbi.nlm.nih.gov/34512170/) | [Q8WZA2](https://www.uniprot.org/uniprot/Q8WZA2) | Target of PDAC released micrRNA-19a, may have role in insulin secretion dysregulation |
| 55 | Proprotein convertase subtilisin/kexin type 1/3 (PCSK1/3)* | PCSK1 | [24] | [29800682](https://pubmed.ncbi.nlm.nih.gov/29800682/) | [P29120](https://www.uniprot.org/uniprot/P29120) | Down regulated by PDAC produced exosomes, which results in decreased GIP levels which are associated with NOD |
| 56 | **Apolipoprotein A-IV** (APOA4) | APOA4 | [25] | [32545216](https://pubmed.ncbi.nlm.nih.gov/32545216/) | [P06727](https://www.uniprot.org/uniprot/P06727) | Reduced plasma levels in patients with PDAC-DM |
| 57 | C-type lectin domain family 3 member B (CLEC3B) | CLEC3B | [25] | [32545216](https://pubmed.ncbi.nlm.nih.gov/32545216/) | [P05452](https://www.uniprot.org/uniprot/P05452) | Reduced plasma levels in patients with PDAC-DM |
| 58 | **Gelsolin** (GSN) | GSN | [25] | [32545216](https://pubmed.ncbi.nlm.nih.gov/32545216/) | [P06396](https://www.uniprot.org/uniprot/P06396) | Reduced plasma levels in patients with PDAC-DM |
| 59 | **Pigment epithelium-derived factor (**SERPINF1) | SERPINF1 | [25] | [32545216](https://pubmed.ncbi.nlm.nih.gov/32545216/) | [P36955](https://www.uniprot.org/uniprot/P36955) | Reduced plasma levels in patients with PDAC-DM |
| 60 | PI3K |  | [26] | [28710412](https://pubmed.ncbi.nlm.nih.gov/28710412/) | ? | Involved in signalling pathway potentially inhibited by PDAC exosome miRNA, which results in inhibition of glucose intake |
| 61 | FoxO1 | FoxO1 | [26] | [28710412](https://pubmed.ncbi.nlm.nih.gov/28710412/) | [Q12778](https://www.uniprot.org/uniprot/Q12778) | Involved in signalling pathway potentially inhibited by PDAC exosome miRNA, which results in inhibition of glucose intake |
| 62 | Akt | AKT1 | [26] | [28710412](https://pubmed.ncbi.nlm.nih.gov/28710412/) | P31749 | Involved in signalling pathway potentially inhibited by PDAC exosome miRNA, which results in inhibition of glucose intake |
| 63 | C-reactive protein (CRP) | CRP | [27] | [25636208](https://pubmed.ncbi.nlm.nih.gov/25636208/) | [P02741](https://www.uniprot.org/uniprot/P02741) | Increased serum levels in patients with PDAC-DM, levels decreased post-surgical resection |
| 64 | TNF super-family member 13 (TNFSF13) | TNFSF13 | [27] | [25636208](https://pubmed.ncbi.nlm.nih.gov/25636208/) | [O75888](https://www.uniprot.org/uniprot/O75888) | Increased serum levels in patients with PDAC-DM, levels decreased post-surgical resection |
| 65 | Kinesin family member 22 (KIF22) | KIF22 | [28] | [34257566](https://pubmed.ncbi.nlm.nih.gov/34257566/) | [Q14807](https://www.uniprot.org/uniprot/Q14807) | Upregulated in patients with PDAC-DM, decreased levels associated with good survival outcomes |
| 66 | Glycogen phosphorylase L (PYGL) | PYGL | [28] | [34257566](https://pubmed.ncbi.nlm.nih.gov/34257566/) | [P06737](https://www.uniprot.org/uniprot/P06737) | Upregulated in patients with PDAC-DM, decreased levels associated with good survival outcomes |
| 67 | ATP-binding cassette 1  (ABCA1) | ABCA1 | [2] | [20571492](https://pubmed.ncbi.nlm.nih.gov/20571492/) | [O95477](https://www.uniprot.org/uniprot/O95477) | Upregulated in patients with PDAC-DM |
| 68 | Arginase-1 (ARG1) | ARG1 | [2] | [20571492](https://pubmed.ncbi.nlm.nih.gov/20571492/) | [P05089](https://www.uniprot.org/uniprot/P05089) | Upregulated in patients with PDAC-DM |
| 69 | CAMP* | RAPGEF3 | [2] | [20571492](https://pubmed.ncbi.nlm.nih.gov/20571492/) | [O95398](https://www.uniprot.org/uniprot/O95398) | Upregulated in patients with PDAC-DM |
| 70 | CD58 | CD8 | [2] | [20571492](https://pubmed.ncbi.nlm.nih.gov/20571492/) | [P19256](https://www.uniprot.org/uniprot/P19256) | Upregulated in patients with PDAC-DM |
| 71 | Carcinoembryonic antigen-related cell adhesion molecule 6 (CEACAM6) | CEACAM6 | [2] | [20571492](https://pubmed.ncbi.nlm.nih.gov/20571492/) | [P40199](https://www.uniprot.org/uniprot/P40199) | Upregulated in patients with PDAC-DM |
| 72 | FLJ22662 |  | [2] | [20571492](https://pubmed.ncbi.nlm.nih.gov/20571492/) | ? | Upregulated in patients with PDAC-DM |
| 73 | LTF | LTF | [2] | [20571492](https://pubmed.ncbi.nlm.nih.gov/20571492/) | [P02788](https://www.uniprot.org/uniprot/P02788) | Upregulated in patients with PDAC-DM |
| 74 | PGK1 | PGK1 | [2] | [20571492](https://pubmed.ncbi.nlm.nih.gov/20571492/) | [P00558](https://www.uniprot.org/uniprot/P00558) | Upregulated in patients with PDAC-DM |
| 75 | CD117 | KIT | [2] | [20571492](https://pubmed.ncbi.nlm.nih.gov/20571492/) | [P10721](https://www.uniprot.org/uniprot/P10721) | Upregulated in patients with PDAC-DM |

Keywords: PDAC, Pancreatic Cancer, Pancreatitis, Diabetes, Diabetes Mellitus, Type 2 Diabetes, New Onset Diabetes, Type 3c Diabetes Mellitus

1. Aggarwal, G., et al., *Adrenomedullin is up-regulated in patients with pancreatic cancer and causes insulin resistance in β cells and mice.* Gastroenterology, 2012. **143**(6): p. 1510-1517.e1.

2. Huang, H., et al., *Novel blood biomarkers of pancreatic cancer-associated diabetes mellitus identified by peripheral blood-based gene expression profiles.* Am J Gastroenterol, 2010. **105**(7): p. 1661-9.

3. Shi, W., et al., *Osteoprotegerin is up-regulated in pancreatic cancers and correlates with cancer-associated new-onset diabetes.* Biosci Trends, 2014. **8**(6): p. 322-6.

4. Basso, D., et al., *Pancreatic cancer-derived S-100A8 N-terminal peptide: a diabetes cause?* Clin Chim Acta, 2006. **372**(1-2): p. 120-8.

5. Jenkinson, C., et al., *Decreased Serum Thrombospondin-1 Levels in Pancreatic Cancer Patients Up to 24 Months Prior to Clinical Diagnosis: Association with Diabetes Mellitus.* Clin Cancer Res, 2016. **22**(7): p. 1734-1743.

6. Susanto, H., et al., *Increased serum levels of betatrophin in pancreatic cancer-associated diabetes.* Oncotarget, 2016. **7**(27): p. 42330-42339.

7. Guo, Q., et al., *Elevated levels of CA 19-9 and CEA in pancreatic cancer-associated diabetes.* J Cancer Res Clin Oncol, 2010. **136**(11): p. 1627-31.

8. Wang, L., et al., *Diabetes mellitus stimulates pancreatic cancer growth and epithelial-mesenchymal transition-mediated metastasis via a p38 MAPK pathway.* Oncotarget, 2016. **7**(25): p. 38539-38550.

9. Ma, X., et al., *Transforming growth factor-β signaling, a potential mechanism associated with diabetes mellitus and pancreatic cancer?* J Cell Physiol, 2020. **235**(9): p. 5882-5892.

10. Suzuki, H., et al., *Effect of insulin-like growth factor gene polymorphisms alone or in interaction with diabetes on the risk of pancreatic cancer.* Cancer Epidemiol Biomarkers Prev, 2008. **17**(12): p. 3467-73.

11. Pfeffer, F., et al., *Expression of connexin26 in islets of Langerhans is associated with impaired glucose tolerance in patients with pancreatic adenocarcinoma.* Pancreas, 2004. **29**(4): p. 284-90.

12. Ding, X., et al., *Pancreatic cancer cells selectively stimulate islet beta cells to secrete amylin.* Gastroenterology, 1998. **114**(1): p. 130-8.

13. Oldfield, L., et al., *Blood levels of adiponectin and IL-1Ra distinguish type 3c from type 2 diabetes: Implications for earlier pancreatic cancer detection in new-onset diabetes.* EBioMedicine, 2022. **75**: p. 103802.

14. Goldberg, R., et al., *Regulation of Heparanase in Diabetes-Associated Pancreatic Carcinoma.* Front Oncol, 2019. **9**: p. 1405.

15. Sun, Y., et al., *Role of transgelin-2 in diabetes-associated pancreatic ductal adenocarcinoma.* Oncotarget, 2017. **8**(30): p. 49592-49604.

16. Zechner, D., et al., *Impact of diabetes type II and chronic inflammation on pancreatic cancer.* BMC Cancer, 2015. **15**: p. 51.

17. Capello, M., et al., *CES2 Expression in Pancreatic Adenocarcinoma Is Predictive of Response to Irinotecan and Is Associated With Type 2 Diabetes.* JCO Precis Oncol, 2020. **4**: p. 426-436.

18. Busek, P., et al., *Increased tissue and circulating levels of dipeptidyl peptidase-IV enzymatic activity in patients with pancreatic ductal adenocarcinoma.* Pancreatology, 2016. **16**(5): p. 829-38.

19. Zhou, L., et al., *Upregulation of REG Ialpha accelerates tumor progression in pancreatic cancer with diabetes.* Int J Cancer, 2010. **127**(8): p. 1795-803.

20. Osman, M.A., F.H. Sarkar, and E. Rodriguez-Boulan, *A molecular rheostat at the interface of cancer and diabetes.* Biochim Biophys Acta, 2013. **1836**(1): p. 166-76.

21. Škrha, J., et al., *Lower plasma levels of glucose-dependent insulinotropic peptide (GIP) and pancreatic polypeptide (PP) in patients with ductal adenocarcinoma of the pancreas and their relation to the presence of impaired glucoregulation and weight loss.* Pancreatology, 2017. **17**(1): p. 89-94.

22. Liao, W.C., et al., *Galectin-3 and S100A9: Novel Diabetogenic Factors Mediating Pancreatic Cancer-Associated Diabetes.* Diabetes Care, 2019. **42**(9): p. 1752-1759.

23. Pang, W., et al., *Pancreatic cancer-derived exosomal microRNA-19a induces β-cell dysfunction by targeting ADCY1 and EPAC2.* Int J Biol Sci, 2021. **17**(13): p. 3622-3633.

24. Zhang, Y., et al., *Pancreatic cancer-derived exosomes suppress the production of GIP and GLP-1 from STC-1 cells in vitro by down-regulating the PCSK1/3.* Cancer Lett, 2018. **431**: p. 190-200.

25. Peng, H., et al., *Systemic Proteome Alterations Linked to Early Stage Pancreatic Cancer in Diabetic Patients.* Cancers (Basel), 2020. **12**(6).

26. Wang, L., et al., *Exosomes derived from pancreatic cancer cells induce insulin resistance in C2C12 myotube cells through the PI3K/Akt/FoxO1 pathway.* Sci Rep, 2017. **7**(1): p. 5384.

27. Gao, W., et al., *Analysis of global gene expression profiles suggests a role of acute inflammation in type 3C diabetes mellitus caused by pancreatic ductal adenocarcinoma.* Diabetologia, 2015. **58**(4): p. 835-44.

28. Zhou, W., et al., *Identification of Key Genes Involved in Pancreatic Ductal Adenocarcinoma with Diabetes Mellitus Based on Gene Expression Profiling Analysis.* Pathol Oncol Res, 2021. **27**: p. 604730.
