# Supplementary figures and images for: Current paradigm and futuristic vision on new-onset diabetes and pancreatic cancer research
Source: Front Pharmacol. 2025 May 23;16:1543112. doi: 10.3389/fphar.2025.1543112 (PMC12141227; doi:10.3389/fphar.2025.1543112)

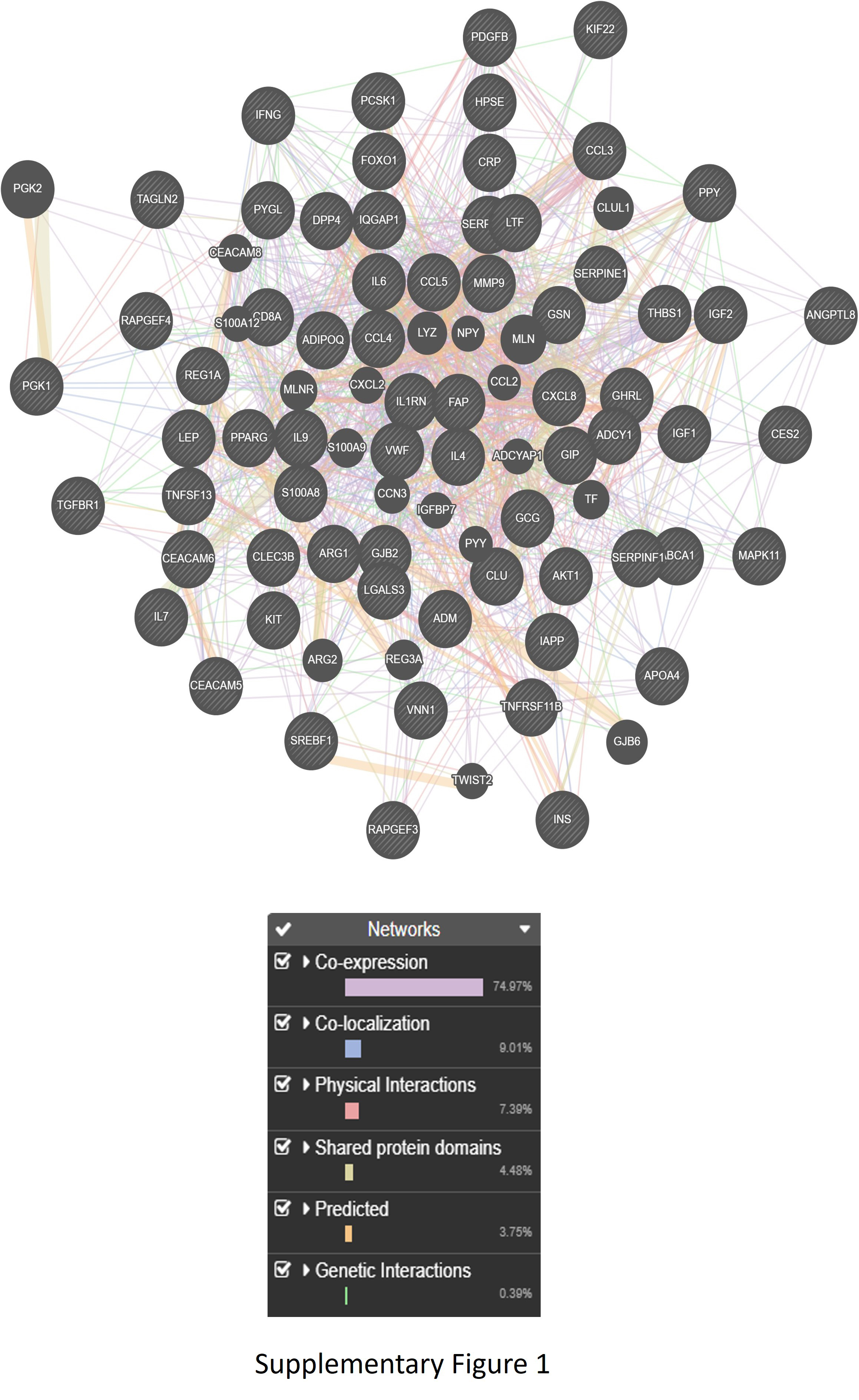

Supplement: Supplementary file 2 [file Image1.jpeg]
