# Supplementary material for: Current paradigm and futuristic vision on new-onset diabetes and pancreatic cancer research
Source: Front Pharmacol. 2025 May 23;16:1543112. doi: 10.3389/fphar.2025.1543112 (PMC12141227; doi:10.3389/fphar.2025.1543112)
Supplement: Supplementary file 3 [file Table2.docx]

**Supplementary Table 2: Pathway and Process Enrichment Analysis of Input Gene Set**

| **Group ID** | **Category** | **GO:ID** | **Pathway & Process** | **LogP** | **Log(q-value)** | **Involvement of genes out of total genes in particular group** | **Contributed Genes** |
| --- | --- | --- | --- | --- | --- | --- | --- |
| 1_Member | Canonical Pathways | M5885 | NABA MATRISOME ASSOCIATED | -22.4828 | -18.135 | 25/751 | SERPINA3,IFNG,IGF1,IGF2,IL1RN,IL4,IL6,IL7,CXCL8,IL9,INS,LEP,LGALS1, LGALS3,MMP9,SERPINE1,PDGFB,SERPINF1,REG1A,S100A8,CCL3,CCL4,CCL5,CLEC3B,TNFSF13,HPSE |
| 2_Member | GO Biological Processes | GO:0001934 | positive regulation of protein phosphorylation | -18.574 | -14.703 | 22/741 | AKT1,CLU,GCG,IFNG,IGF1,IGF2,IL4,IL6,INS,KIT,LEP,LTF,MMP9,PDGFB,PPARG,CCL5,TGFBR1,THBS1,IQGAP1,ADIPOQ,RAPGEF3,GHRL |
| 3_Member | GO Biological Processes | GO:0009725 | response to hormone | -17.1408 | -13.492 | 21/753 | ADM,AKT1,ARG1,FOXO1,GIP,GJB2,IGF2,IL1RN,IL6,INS,KIT,LEP,TNFRSF11B,PCSK1,SERPINF1,PPARG,REG1A,SREBF1,THBS1,ADIPOQ,GHRL |
| 4_Member | GO Biological Processes | GO:0062013 | positive regulation of small molecule metabolic process | -16.9152 | -13.345 | 13/149 | ADM,AKT1,APOA4,FOXO1,GCG,IFNG,IGF1,IGF2,IL4,INS,PPARG,SREBF1,ADIPOQ |
| 5_Member | GO Biological Processes | GO:0030155 | regulation of cell adhesion | -15.5303 | -12.296 | 20/786 | AKT1,ARG1,DPP4,IFNG,IGF1,IGF2,IL1RN,IL4,IL6,IL7,CXCL8,LEP,LGALS3,CEACAM6,SERPINE1,PDGFB,CCL5,THBS1,VNN1,ADIPOQ |
| 6_Member | GO Biological Processes | GO:0043065 | positive regulation of apoptotic process | -14.7945 | -11.725 | 17/529 | ADM,CLU,FAP,FOXO1,GSN,IAPP,IFNG,IL6,LEP,MMP9,PPARG,S100A8,CCL3,CCL5,TGFBR1,THBS1,ADIPOQ |
| 7_Member | GO Biological Processes | GO:2001234 | negative regulation of apoptotic signaling pathway | -14.4389 | -11.453 | 13/230 | AKT1,CLU,IGF1,IL4,IL7,IL9,INS,LGALS3,MMP9,SERPINE1,TGFBR1,THBS1,VNN1 |
| 8_Member | GO Biological Processes | GO:0045596 | negative regulation of cell differentiation | -14.0207 | -11.088 | 18/699 | ABCA1,CEACAM5,CRP,FOXO1,IAPP,IFNG,IGF1,IGF2,IL4,IL6,LTF,MMP9,TNFRSF11B,PDGFB,PPARG,CCL3,TGFBR1,ADIPOQ |
| 9_Member | GO Biological Processes | GO:0010817 | regulation of hormone levels | -13.9126 | -11.012 | 16/498 | ADM,DPP4,GCG,GIP,IFNG,IL1RN,IL6,INS,LEP,PCSK1,PPARG,CCL5,SREBF1,ADIPOQ,RAPGEF4,GHRL |
| 10_Member | Reactome Gene Sets | R-HSA-76002 | Platelet activation, signaling and aggregation | -13.6867 | -10.857 | 13/263 | SERPINA3,AKT1,CLU,IGF1,IGF2,SERPINE1,PDGFB,THBS1,CLEC3B,VWF,TAGLN2,RAPGEF3,RAPGEF4 |
| 11_Member | GO Biological Processes | GO:0019216 | regulation of lipid metabolic process | -13.6205 | -10.804 | 14/341 | SERPINA3,ADM,AKT1,APOA4,GIP,IFNG,INS,KIT,LEP,PDGFB,PPARG,SREBF1,ADIPOQ,ANGPTL8 |
| 12_Member | KEGG Pathway | hsa05142 | Chagas disease | -13.5852 | -10.781 | 10/102 | ADCY1,AKT1,IFNG,IL6,CXCL8,SERPINE1,MAPK11,CCL3,CCL5,TGFBR1 |
| 13_Member | GO Biological Processes | GO:0001775 | cell activation | -13.3686 | -10.589 | 17/647 | CD8A,CLU,DPP4,IFNG,IGF1,IL4,IL6,IL7,CXCL8,IL9,INS,KIT,LEP,CCL3,CCL5,TGFBR1,VWF |
| 14_Member | GO Biological Processes | GO:0050727 | regulation of inflammatory response | -12.6751 | -9.960 | 14/400 | IFNG,IGF1,IL4,IL6,INS,MMP9,SERPINE1,SERPINF1,PPARG,S100A8,CCL3,CCL5,ADIPOQ,GHRL |
| 15_Member | WikiPathways | WP1533 | Vitamin B12 metabolism | -12.5059 | -9.821 | 8/53 | SERPINA3,ABCA1,CRP,IFNG,IL6,INS,SERPINE1,CCL5 |
| 16_Member | GO Biological Processes | GO:0010035 | response to inorganic substance | -12.3526 | -9.721 | 15/521 | ADCY1,AKT1,APOA4,ARG1,FOXO1,GIP,IL6,KIT,MMP9,TNFRSF11B,PCSK1,SERPINF1,S100A8,THBS1,IQGAP1 |
| 17_Member | GO Biological Processes | GO:0009617 | response to bacterium | -11.3663 | -8.867 | 16/732 | ABCA1,ADM,AKT1,ARG1,CRP,GJB2,IL6,CXCL8,LTF,SERPINE1,PCSK1,PYGL,S100A8,CCL3,CCL5,ADIPOQ |
| 18_Member | GO Biological Processes | GO:0030162 | regulation of proteolysis | -11.2518 | -8.779 | 16/745 | SERPINA3,AKT1,CLU,GSN,IFNG,IL9,INS,LTF,MMP9,SERPINE1,SERPINF1,PPARG,S100A8,THBS1,CLEC3B,ANGPTL8 |
| 19_Member | GO Biological Processes | GO:1905952 | regulation of lipid localization | -11.0181 | -8.599 | 10/183 | ABCA1,AKT1,APOA4,CRP,IL6,LEP,PPARG,THBS1,ADIPOQ,GHRL |
| 20_Member | GO Biological Processes | GO:0009611 | response to wounding | -10.9186 | -8.515 | 13/435 | ADM,APOA4,ARG1,GIP,IGF1,IL6,INS,PCSK1,PDGFB,S100A8,TGFBR1,VWF,HPSE |
