# Supplementary material for: Current paradigm and futuristic vision on new-onset diabetes and pancreatic cancer research
Source: Front Pharmacol. 2025 May 23;16:1543112. doi: 10.3389/fphar.2025.1543112 (PMC12141227; doi:10.3389/fphar.2025.1543112)
Supplement: Supplementary file 4 [file Table3.docx]

| **Supplementary Table 3:** PC risk in diverse ethnic groups | |
| --- | --- |
| **Ethnic Group** | **Pancreatic Cancer Risk Factors** |
| Black | Genetic predisposition, increased prevalence of Type 2 diabetes, disparities in healthcare access and smoking (Silverman et al., 2003;Huang et al., 2019;Vick et al., 2019). |
| Hispanic/Latino | Higher rates of insulin resistance, obesity, and metabolic syndrome and limited access to healthcare and preventive services (Huang et al., 2019). |
| Asian | Unique genetic factors affecting diabetes progression, lower BMI despite metabolic dysfunction (Xiang et al., 2023) (Zhao et al., 2023). |
| Native/Indiginious | Metabolic differences, dietary factors, and central obesity (Kills First et al., 2022). |

LITERATURE CITED

Guadagnolo, B.A., Petereit, D.G., and Coleman, C.N. (2017). Cancer Care Access and Outcomes for American Indian Populations in the United States: Challenges and Models for Progress. *Semin Radiat Oncol* 27**,** 143-149.

Huang, B.Z., Stram, D.O., Le Marchand, L., Haiman, C.A., Wilkens, L.R., Pandol, S.J., Zhang, Z.F., Monroe, K.R., and Setiawan, V.W. (2019). Interethnic differences in pancreatic cancer incidence and risk factors: The Multiethnic Cohort. *Cancer Med* 8**,** 3592-3603.

Kills First, C.C., Sutton, T.L., Shannon, J., Brody, J.R., and Sheppard, B.C. (2022). Disparities in pancreatic cancer care and research in Native Americans: Righting a history of wrongs. *Cancer* 128**,** 1560-1567.

Silverman, D.T., Hoover, R.N., Brown, L.M., Swanson, G.M., Schiffman, M., Greenberg, R.S., Hayes, R.B., Lillemoe, K.D., Schoenberg, J.B., Schwartz, A.G., Liff, J., Pottern, L.M., and Fraumeni, J.F., Jr. (2003). Why do Black Americans have a higher risk of pancreatic cancer than White Americans? *Epidemiology* 14**,** 45-54.

Vick, A.D., Hery, D.N., Markowiak, S.F., and Brunicardi, F.C. (2019). Closing the Disparity in Pancreatic Cancer Outcomes: A Closer Look at Nonmodifiable Factors and Their Potential Use in Treatment. *Pancreas* 48**,** 242-249.

Xiang, X., Chen, X., He, Y., Wang, Y., Xia, W., Ye, S., Wang, S., Xiao, Y., Li, Q., Wang, X., Luo, W., and Li, J. (2023). Pancreatic cancer challenge in 52 Asian countries: age-centric insights and the role of modifiable risk factors (1990-2019). *Front Oncol* 13**,** 1271370.

Zhao, Z., Li, X., Wang, F., Xu, Y., Liu, S., Han, Q., Yang, Z., Huang, W., Yin, Z., Liu, Q., Tan, H., Ma, T., Si, S., Huang, J., Yuan, H., Li, W., and Liu, R. (2023). Pathogenic genomic alterations in Chinese pancreatic cancer patients and their therapeutical implications. *Cancer Med* 12**,** 11672-11685.
